# Association of Neuronal Autoantibodies with Overall Survival in Patients with Gastric Cancer

**DOI:** 10.1158/2767-9764.CRC-25-0495

**Published:** 2025-12-03

**Authors:** Guanghui Liu, Xin Tan, Kaibiao Xu, Jingsi Liu, Qianhui Zheng, Xiaoyun Huang, Suyue Pan, Guoxin Li, Yafang Hu, Hao Liu

**Affiliations:** 1 Department of Neurology, Nanfang Hospital, Southern Medical University, Guangzhou, China.; 2 Department of General Surgery, Nanfang Hospital, Southern Medical University, Guangzhou, China.; 3 JC School of Public Health and Primary Care, Faculty of Medicine, The Chinese University of Hong Kong, Hong Kong SAR, China.; 4 Cancer Center of Beijing Tsinghua Changgung Hospital, School of Clinical Medicine, Tsinghua Medicine, Tsinghua University, Beijing, China.

## Abstract

Neuronal autoantibodies have been identified in immune-mediated encephalitis, most of which are related to paraneoplastic neurologic syndromes (PNS). We detected neuronal autoantibodies in patients with gastric cancer without PNS and illustrated their correlation with clinical prognosis. All serum samples were tested by the mouse brain tissue-based assay (TBA) using immunofluorescence for screening neuronal autoantibodies. Known PNS-related neuronal autoantibodies were detected by cell-based assay. A single-center cohort has been started in Nanfang Hospital. The T-cell status of the tumor microenvironment was assessed. Single-cell sequencing was performed with limited tumor samples. Patients were grouped into TBA-positive (*n* = 144) and TBA-negative (*n* = 179) groups by the TBA status. Further screening of TBA+ specimens using the cell-based assay method revealed known PNS-related autoantibodies in 13.2% (19/144) of cases. Additionally, TBA-positive patients with gastric cancer exhibited lower CD8^+^ T-cell infiltration in the tumor tissue. The survival analysis show that neuronal autoantibodies in patients (TBA-positive) were associated with shorter overall survival (OS; *P* = 0.014). In the multivariate survival analysis, TBA positivity was still associated with shortened OS after adjusting the major covariates (HR = 2.28; 95% confidence interval, 1.31–3.97; *P* = 0.004). Meanwhile, single-cell sequencing indicates that cell junction assembly and synapse organization may play important roles in biological process. In this cohort study, neuronal autoantibodies were highly prevalent among patients with gastric cancer and were associated with shortened OS and features of immunosuppression within the tumor microenvironment, suggesting a candidate for exploring therapeutic relevance, with further mechanistic studies needed for validation.

**Significance:**

Neuronal autoantibodies are prevalent in patients with gastric cancer, and patients without neurologic symptoms are linked to shorter survival and immunosuppression. These results provide a new direction for prognostic biomarkers and targeted therapy exploration.

## Introduction

The relationship between tumors and neuronal autoantibodies in the peripheral blood of patients with cancer has gained significant attention in recent years, particularly outside the context of paraneoplastic neurologic syndromes (PNS). This emerging field, situated at the intersection of neuro-oncology and immunology, underscores the importance of understanding how neuronal antibodies can influence cancer prognosis and patient outcomes. Neuronal autoantibodies usually targeting intracellular components of the neuronal cell are first found in PNS (1). These syndromes manifest when an immune response against a tumor inadvertently targets the nervous system, leading to neurologic complications (2, 3). However, emerging evidence suggests that neuronal antibodies are present in the peripheral blood of patients with cancer without apparent PNS (4, 5). The origin and function of these nonparaneoplastic neuronal antibodies remain incompletely understood. They may arise from immune responses targeting tumors expressing onconeuronal antigens or undergoing neuronal differentiation or potentially play direct roles in the neuronal tumor-immune microenvironment. In addition, neuronal autoantibodies directed against cell-surface or synaptic proteins define entities such as limbic encephalitis and anti-N-Methyl-D-Aspartate Receptor (anti-NMDAR) encephalitis; these conditions may, but do not always, reflect tumor-associated autoimmunity.

The presence of neuronal autoantibodies may reflect an ongoing immune response to the tumor itself, offering a potential biomarker for cancer progression and patient prognosis. Understanding the clinical significance of neuronal antibodies in patients with cancer involves exploring their potential roles in tumor biology. A review on paraneoplastic neuropathies emphasizes that onconeural antibodies (e.g., anti-Hu and Caspr2) mediate a complex cross-talk between the immune system and tumor microenvironment (TME), potentially modulating cancer growth, metastasis, and therapeutic response by targeting shared antigens in neurons and tumor cells (6). Moreover, the detection of neuronal antibodies could aid in the early diagnosis of cancer, monitor disease progression, and predict therapeutic outcomes. A case report first documents the association between gastric adenocarcinoma and anti–γ-aminobutyric acid-B receptor antibody-related limbic encephalitis, suggesting that anti–γ-aminobutyric acid-B receptor antibody detection may have auxiliary diagnostic value for gastric adenocarcinoma (7). Investigating the relationship between neuronal antibodies and clinical outcomes in patients with cancer holds substantial implications for personalized medicine. Elucidating how these antibodies influence tumor behavior could enable the development of targeted therapies that modulate the immune response, potentially improving both survival and quality of life. Recent studies have highlighted that neuronal autoantibodies can be associated with significant cognitive impairment in patients with cancer, indicating a broader impact on patient health beyond tumor progression (8, 9).

In addition to their potential as biomarkers, neuronal antibodies are increasingly recognized for their roles in modulating the TME. For instance, tumor cells can interact with and modify the behavior of peripheral nerves, which in turn can affect tumor growth and metastasis (10). This neuroimmune interaction is a growing area of interest, as it may uncover new therapeutic targets that leverage the nervous system to combat cancer. Thus, the study of neuronal antibodies in patients with cancer without PNS represents a critical area of research that promises to enhance our understanding of cancer immunology and pave the way for novel diagnostic and therapeutic strategies. The tumor and central nervous system immunity are closely related, but there are few reports on the neuroimmune system in gastric cancer. To address this gap, this study analyzed the characteristics of antineuronal autoantibodies in patients with gastric cancer. In present study, we aimed to assess the prevalence of neuronal autoantibodies in patients with gastric cancer and their association with prognosis in a cohort study. We therefore (i) screened a large cohort of patients with gastric cancer for neuronal autoantibodies, (ii) compared prognosis between antibody-positive and antibody-negative patients, and (iii) investigated the impact of neuronal autoantibodies on tumor-immune microenvironment.

## Materials and Methods

### Patient characteristics

A total of 323 consecutive newly diagnosed patients with gastric cancer were recruited at the Department of General Surgery, Nanfang Hospital, Southern Medical University, Guangzhou, China, between November 2021 and July 2023. All enrolled participants finished the study, i.e., there was no attrition. Inclusion criteria: Patients with a histologically confirmed diagnosis of gastric cancer. Patients with previous chemotherapy or diagnosed PNS were excluded from this study. Baseline demographic and clinical characteristics, including age, sex, body mass index, Lauren classification, treatment regimen, histologic type, PD-L1 status, HER2 status, and cancer stage were obtained from medical records.

### Neuronal autoantibody analysis

Serum samples from the patients were analyzed using well-established assays, including cell-based assay (CBA) and mouse brain tissue-based assay (TBA) using immunofluorescence staining. Autoantibody testing was performed by investigators (including G.L) who were blinded to the survival outcomes. Briefly, TBA kits were prepared as previously reported (11, 12): brains from 3-month-old male C57BL/6J mice (Risemice Biotechnology Co., Ltd.) were sectioned sagittally at a thickness of 5 μm, fixed with cold acetone for 10 minutes, and stored at −20°C before use. CBA kits were used for PNS autoantibodies’ screening, which were prepared by transfected pcDNA3.1 plasmids expressing the targeting antigens in HEK-293T cells, respectively, including full-length human cDNA of Hu (NM_001324208), Yo (NM_020134), Ri (NM_002515 and NM_002516, cotransfected), Ma2 (NM_007257), CV2/CRMP5 (NM_020134), amphiphysin (NM_001635), Tr (DNER; NM_139072), SOX1 (NM_005986), PCA2 (NM_005909), Zic4 (NM_005909), KLHL11 (NM_018143), Titin (NM_001256850, MIR region), Ma1 (NM_006029), recoverin (NM_002903), PKCγ (NM_001316329), and GAD65 (NM_000818). The HEK-293T cell line, verified by short tandem repeat sequencing, was purchased from the China Center for Type Culture Collection (cat. #SCSP-502) and kept in our laboratory. The cells were cultured with 10% FBS (Gibco, A5256701) in DMEM (Gibco, C11960500BT) at 37°C, 5% CO_2_, and tested for Mycoplasma weekly. The cells of normal morphology and passaged less than 20 times after thawing were used for transfection with Lipo8000 (Beyotime, C0533) according to the instructions. All the plasmids were constructed by GenScript and verified by sequencing. The transfected cells were fixed with 4% paraformaldehyde in Phosphate Buffered Saline (PBS) for 15 minutes and washed with PBS. For immunostaining with gastric cancer tissues, paraffin sections of the biopsies from the patients were prepared and retrieved by incubation in Tris-EDTA (pH 9.0) at 95 to 100°C for 15 minutes. The sections (TBA and gastric cancer tissues) and CBA cells were permeated with 0.3% TritonX-100 in PBS for 10 minutes and then blocked with 1% BSA in Phosphate Buffered Saline with Tween (PBST) for 1 hour at room temperature (RT). Serum samples (diluted at 1:100 with 1% BSA in PBST), anti-CD4 (Proteintech, 19068-1-AP, 1:300), and CD8 (Proteintech, 29896-1-AP, 1:300) antibodies were added as needed and incubated for 1 hour at RT. After three times washing with PBST, appropriate secondary antibodies, goat anti-human IgG-Alexa Fluor 488 (Thermo Fisher Scientific, A11013, 1:1,000) or goat anti-rabbit IgG-Alexa Fluor 594 (Thermo Fisher Scientific, A11012, 1:1,000), were added and incubated for 1 hour at RT. The sections and cells were washed and sealed with mounting solution containing 4′,6′-Diamidino-2′-phenylindole (Beyotime, P0131). The images were taken using inverted fluorescent microscope (Olympus, IX73 or Nikon, Ti2). To quantify the CD4^+^ and CD8^+^ cells in gastric cancer tissues, the cells were counted manually for at least five random fields per section under a 40× objective magnification.

### Single-cell sequencing

Fresh tissue was digested with collagenase II/IV + dispase, filtered (40 μm), and purified by red blood cell lysis, and viability was checked using Cellometer Auto 2000 (AO/PI). Libraries made with 10x Genomics Chromium Next GEM version 3.1 and sequenced on Illumina NovaSeq 6000 (PE150, ∼500M reads/library). Raw data aligned (GRCh38) with Cell Ranger version 7.1.0; Seurat retained cells (100 <nFeature_RNA <2,000, nCount_RNA <10,000, percent.mt <20%). Samples were integrated via Canonical Correspondence Analysis, principal component analysis/Uniform Manifold Approximation and Projection for reduction, and Shared Nearest Neighbor clustering (resolution 0.5); cell types were annotated by the top 10 cluster markers (log fold change ≥0.25). Differentially expressed genes were identified via Seurat (Wilcoxon test, adj. *P* < 0.05); Gene Ontology Biological Process (GOBP) enrichment was performed with clusterProfiler version 4.0.5 (Benjamini–Hochberg correction, *P* < 0.01).

### Statistical analysis

Baseline characteristics were summarized using means (SD) for continuous variables and frequencies (percentages) for categorical variables. Comparisons between groups were made using Welch *t* test for continuous variables and Pearson *χ*^2^ test for categorical variables. Kaplan–Meier survival curves were used to estimate overall survival (OS) probabilities, and differences between groups were evaluated using the log-rank test. The OS probabilities were calculated from the date of diagnosis to the date of death or last follow-up. Survival curves were generated for the specific patients according to the TBA and CBA statuses. Cox proportional hazards regression was performed to assess the impact of various covariates on OS. HRs and 95% confidence intervals (CI) were calculated for each variable. For GOBP analysis, the *P* value cutoff and the q value cutoff were set as 0.05 and 0.05, respectively. The method used for *P* value adjustment is Benjamini–Hochberg analysis. Statistical analysis was performed using IBM SPSS Statistics, version 23 (IBM SPSS, RRID: SCR_002865); data handing and plotting were performed using packages in R software (version 4.2.1, RRID: SCR_001905). Packages “survival” (version 3.5-7), “survminer” (version 0.4.9), and “ggplot2” (version 3.5.1) were used in this analysis.

### Ethical considerations

The prospective study was approved by the ethics committee of Nanfang Hospital, Guangzhou, China (NFEC-2024-247). The study followed the Strengthening the Reporting of Observational Studies in Epidemiology reporting guideline for cohort studies. The mice usage for TBA assays was approved by the Animal Ethics Committee of Nanfang Hospital (IACUC-LAC-20220608-002), and all experiments were conducted in accordance with the institutional guidelines.

## Results

### Baseline characteristics

According to the TBA status, the patients were grouped into TBA-positive (*n* = 144) and TBA-negative (*n* = 179) groups. The mean age was similar between TBA-positive (60.8 years) and TBA-negative (59.5 years) groups (*P* = 0.29). There were similar females in both groups (32.6% vs. 39.7%), with no significant difference (*P* = 0.24). Both groups had similar body mass index distributions (22.4 vs. 22.7, *P* = 0.36). The distribution of Lauren classification (diffuse or mixed vs. intestinal) was not significantly different between the two groups (*P* = 0.42). Also, there was no statistical difference in the treatment modality, pathologic tumor stages, histologic types, and Her2 and PD-L1 expressions between the two groups (Table 1).

**Table 1.** Baseline characteristics of patients with gastric cancer by TBA status.

Variables	TBA-positive (*n* = 144)	TBA-negative (*n* = 179)	*P* value
Age	60.8 (11.2)	59.5 (10.7)	0.294
Body mass index	22.4 (2.9)	22.7 (3.1)	0.359
Female	47 (32.6%)	71 (39.7%)	0.235
Lauren type	​	​	0.417
Diffuse or mixed	68 (74.7%)	88 (68.8%)	​
Intestinal	23 (25.3%)	40 (31.3%)	​
NA	53	51	​
Radical surgery	96 (66.7%)	137 (76.5%)	0.066
Histology	​	​	0.522
Signet ring cell carcinoma	57 (39.9%)	64 (35.8%)	​
Other types	86 (60.1%)	115 (64.2%)	​
NA	1	0	​
Pathologic tumor–node–metastasis	​	​	0.165
IV	46 (31.9%)	40 (22.3%)	​
III	31 (21.5%)	54 (30.2%)	​
II	24 (16.7%)	29 (16.2%)	​
I	43 (29.9%)	56 (31.3%)	​
PD-L1 expression	​	​	0.282
≥5	60 (42.0%)	62 (35.4%)	​
<5	83 (58.0%)	113 (64.6%)	​
NA	1	4	​
HER2 expression	​	​	0.125
++/+++	26 (18.4%)	20 (11.6%)	​
−/+	115 (81.6%)	152 (88.4%)	​
NA	3	7	​

### Frequency of neuronal autoantibodies

**Figure 1.**
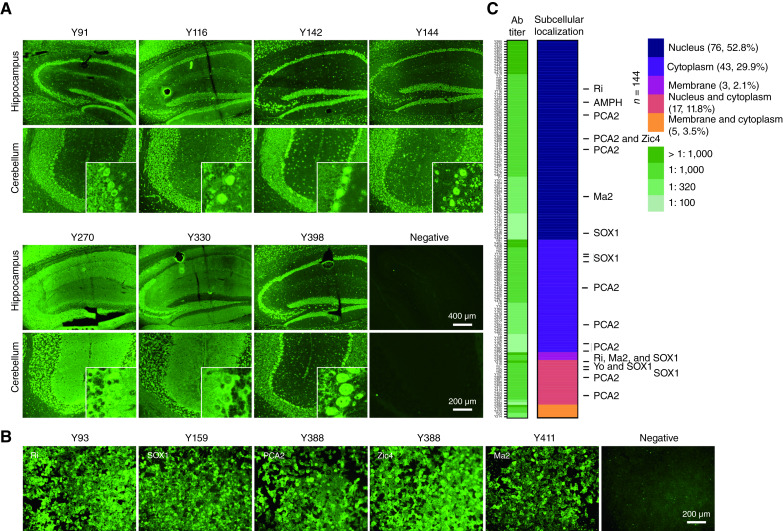
Neuronal autoantibody staining patterns and identification of known PNS-related antibodies in patients with gastric cancer. Ab, antibody.

**Figure 2.**
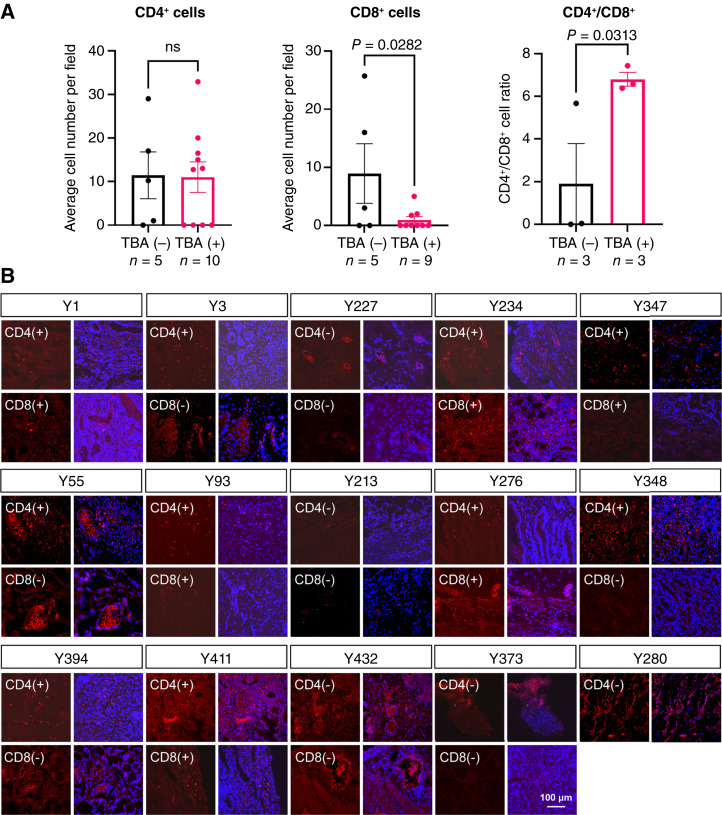
Reduced CD8^+^ T-cell infiltration and altered CD4^+^/CD8^+^ ratio in TBA-positive gastric cancer tissues. ns, not significant.

All serum samples of the 323 patients were first tested using by TBA, a commonly used method for screening neuronal autoantibodies. The results showed that 44.6% (144/323) of the patient samples tested positively for TBA. The staining pattern was primarily intracellular neuronal staining (136/144, 94.4%), which corresponded to the characteristics of the classic PNS. Among them, nuclear staining was observed in 52.8% (76/144), cytoplasmic staining in 29.9% (43/144), and both nuclear and cytoplasmic staining in 11.8% (17/144). Additionally, 2.1% (3/144) of cases exhibited cell membrane staining, and 3.5% (5/144) showed both membrane and cytoplasmic staining (Fig. 1A and B). Further screening of TBA-positive samples using the CBA method revealed known PNS-related autoantibodies in 13.2% (19/144) of cases, including Ri, amphiphysin, SOX1, PCA2, Zic4, Ma2, and Yo (Fig. 1C). Further analysis revealed that, compared with TBA-negative patients, TBA-positive patients exhibited lower CD8^+^ T-cell infiltration in the tumor tissue (0.97 ± 0.57 vs. 8.94 ± 5.13, *P* < 0.05) and a higher CD4^+^/CD8^+^ T-cell ratio (6.80 ± 0.32 vs. 1.90 ± 1.88, *P* < 0.05; Fig. 2), indicating that TBA positivity correlates with an immunosuppressive TME and diminished cytotoxic T-cell activity against tumors.

### Survival analysis

**Figure 3.**
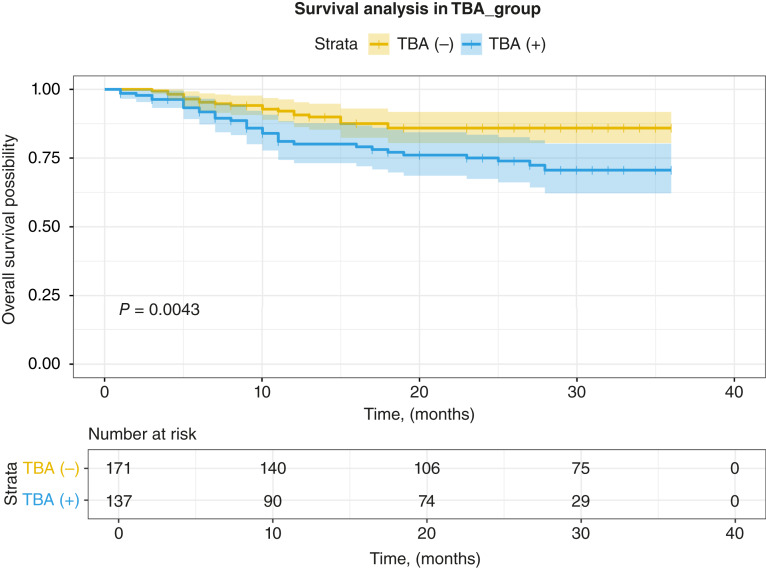
Kaplan–Meier survival curves for OS in TBA-positive and TBA-negative patients with gastric cancer.

**Figure 4.**
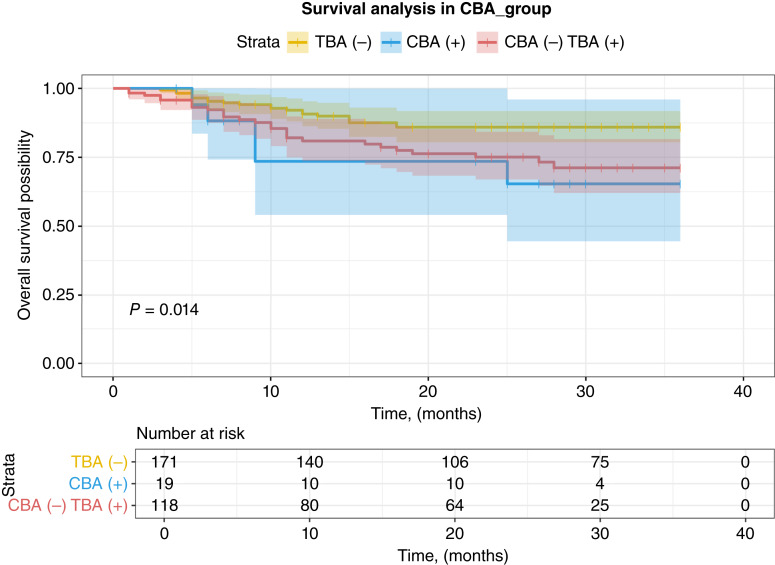
Comparison of 3-year OS among TBA-negative, CBA-positive, and TBA-positive CBA-negative groups.

Because median survival was not reached in each group, we compared the survival probabilities at 36 months. The median follow-up duration is 11 mouths, and all patients included were followed up. As shown in Fig. 3, compared with TBA-negative patients, TBA positivity was associated with poor OS in patients with gastric cancer at 3 years (85.94% vs. 70.60%, *P* = 0.004). We further grouped the patients into three groups taking the CBA result into consideration: TBA−, CBA+, or TBA+ CBA− patients. Comparing the three groups (Fig. 4) revealed statistically significant differences in OS at 3 years between the groups (85.94% vs. 65.40% vs. 71.16%, *P* = 0.014), and the CBA-positive group, which represents having known PNS antibodies, had the worst outcome. The Cox regression model analysis was performed for the HRs for various clinical variables affecting OS (Table 2). Key findings included the following: TBA positivity was associated with shortened OS in patients with gastric cancer (HR = 2.17; 95% CI, 1.25–3.76; *P* = 0.006); surgical treatment significantly reduced the risk of mortality (HR = 0.09; 95% CI, 0.05–0.16; *P* < 0.001). More advanced tumor stage significantly elevated mortality risk (HR = 31.90; 95% CI, 7.75–131.00; *P* < 0.001). In the multivariate analysis, TBA positivity was still associated with shortened OS after adjusting the major covariates (HR = 2.28; 95% CI, 1.31–3.97; *P* = 0.004).

**Table 2.** Cox proportional hazards regression analysis of factors affecting OS in patients with gastric cancer.

Variable	Univariable Cox regression		Multivariable Cox regression	
	HR (95% CIs)	*P* value	HR (95% CIs)	*P* value
Age (years)	1.05 (1.03–1.08)	<0.001	​	​
Sex (female vs. male)	0.83 (0.48–1.44)	0.514	​	​
Body mass index (kg/m^2^)	0.76 (0.38–1.50)	0.424	​	​
Lauren classification (diffuse or mixed type vs. intestinal type)	1.92 (0.56–6.58)	0.301	​	​
Radical surgery (patients with radical surgery vs. patients without surgery)	0.09 (0.05–0.16)	<0.001	​	​
Histologic type (signet ring cell carcinoma vs. others)	0.82 (0.46–1.45)	0.493	​	​
PD-L1 (≥5 vs. <5)	0.63 (0.34–1.17)	0.142	​	​
HER2 (2+/3+ vs. 1+/−)	0.88 (0.41–1.88)	0.739	​	​
Tumor stage (III/IV vs. I/II)	31.90 (7.75–131.00)	<0.001	32.6 (7.92–134.00)	<0.001
TBA (+) vs. TBA (−)	2.17 (1.25–3.76)	0.006	2.28 (1.31–3.97)	0.004

### Single-cell sequencing data results

**Figure 5.**
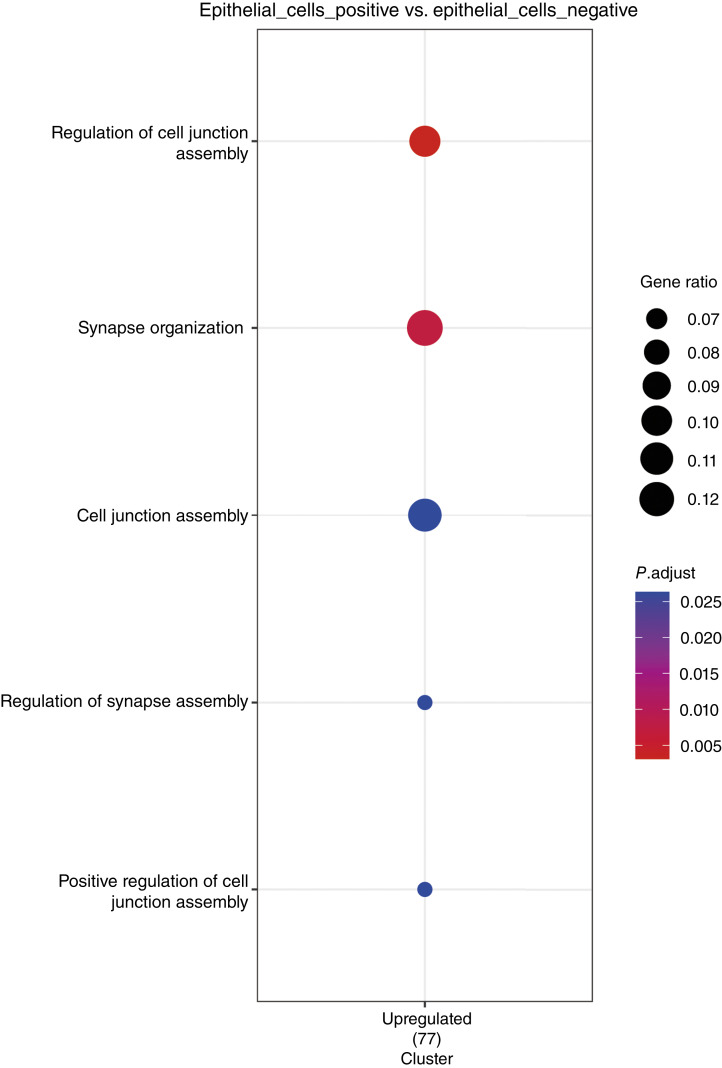
Gene Ontology enrichment analysis of differentially expressed genes in TBA-positive vs. TBA-negative gastric cancer cells.

We performed single-cell transcriptomic analysis on two TBA-negative samples and two TBA-positive samples from four patients with gastric cancer. After quality control, the single-cell atlas of gastric cancer contains a total of 11,422 cells. Overrepresentation analysis was used to enrich pathways associated with positivity of neuronal antibodies. In the comparative analysis of tumor cells (TBA-positive vs. TBA-negative), Gene Ontology enrichment for biological processes revealed significant functional remodeling (Fig. 5). Specifically, functional enrichment of 77 genes significantly upregulated in the TBA+ epithelial cell cluster showed strong enrichment (*P* < 0.05) in biological processes related to cell junction assembly and synapse organization.

## Discussion

This cohort study assessed the prevalence of neuronal autoantibodies and their association with OS in patients with gastric cancer. The findings indicated that 44.6% of patients had neuronal autoantibodies. Among them, only 5.9% had known PNS autoantibodies identified through CBAs, and the remaining had currently unidentified neuronal autoantibodies. However, all patients having neuronal antibodies did not show any neurologic symptoms or signs. Compared with autoantibody-negative patients, patients with neuronal autoantibodies had shortened OS and decreased CD8^+^ T tumor-infiltrating lymphocytes in the TME, implying an affected tumor-immune microenvironment. Limited single-cell sequencing data suggest that cell junction assembly and synapse organization may be involved in biological process.

Neuronal antibodies in patients with cancer have been traditionally studied within the context of PNS, in which the immune response against a tumor mistakenly targets neuronal tissues, leading to various neurologic manifestations (13–15). In current study, we also detected similar neuronal antibodies in patients with gastric cancer without PNS. Recent research has expanded these results, recognizing the presence and potential roles of neuronal antibodies in patients with cancer without evident PNS. This emerging field at the intersection of neuro-oncology and immunology underscores the broader implications of neuronal antibodies in cancer prognosis, progression, and patient outcomes.

The presence of neuronal antibodies in the peripheral blood of patients with cancer poses significant diagnostic and clinical challenges (15). These antibodies can lead to misdiagnosis if not correctly interpreted within the clinical context. For example, the detection of antibodies against intracellular antigens, such as anti-titin and anti-recoverin, can yield false-positive results when using commercial blots assay. This necessitates careful consideration of clinical and paraclinical evidence before diagnosing autoimmune encephalitis or PNS (16). Misinterpretation can be avoided by adhering to stringent diagnostic criteria, corroborating serological findings with clinical symptoms, and utilizing confirmatory testing methods, such as CBA methods, or further cerebrospinal fluid analysis.

Increasing evidence has linked the humoral immune response with the development of various cancers. Human B cells can contribute to tumorigenesis by differentiating into plasma cells and producing various antibodies (17). Studies found an inconsistent association among IgE, IgA, and IgG serum levels in relation to cancer risk (18). Given the diversity antibody types, antibody profiling holds potential for more personalized cancer diagnostics. Among various antibodies, the detection of neuronal antibodies in patients with cancer offers a promising avenue for their use as biomarkers. These antibodies can provide insights into the ongoing immune response against tumors, aiding in early diagnosis, monitoring disease progression, and predicting therapeutic outcomes (15, 19). For instance, amphiphysin-IgG alone is highly associated with breast cancer, but when amphiphysin-IgG is accompanied by Hu (ANNA1)-IgG or CRMP5-IgG, the cancer search is redirected toward lung cancer (20). This broader impact highlights the importance of integrating neuronal antibody testing into routine oncologic diagnostics and patient management strategies.

The cellular immune response plays an equally important role in determining cancer risk and prognosis. In our study, we observed reduced infiltration of CD8^+^ T cells in the TBA+ group. GOBP enrichment analysis of gastric cancer cells identified significant enrichment of synaptic organization and cell junction–related biological processes, suggesting a potential cross-talk between neuronal antibody–mediated immune cell depletion and TME remodeling via neuronal-like functional pathways (21). In our previous study, we also demonstrated that CD8^+^ T-cell abundance is associated with gastric cancer prognosis (22). Based on these findings, we propose that neuronal antibodies may influence the TME, which warrants further investigation. It is already known that tumor cells can interact with peripheral nerves, modifying their behavior and influencing cancer growth and metastasis (23, 24). This neuroimmune interaction is a critical area of research, providing a foundation for exploring potential new therapeutic targets that leverage the nervous system to address cancer. For instance, neural invasion is now considered a fourth route of metastasis, with tumor cells hijacking neural pathways to facilitate their spread (25). We believe the neuronal antibody is a new route to explain the interaction between the nervous and immune systems. Understanding these interactions opens up new possibilities for targeted therapies that disrupt these pathologic neuroimmune communications.

Tumor immune escape is an important strategy of tumor survival. There are many mechanisms of tumor immune escape, including immunosuppression, which has become a research hotspot in recent years. The PD-L1/PD-1 signaling pathway is an important component of tumor immunosuppression, which can inhibit the activation of T-cell lymphocytes and enhance the immune tolerance of tumor cells, thereby achieving tumor immune escape (26, 27). Therefore, targeting the PD-L1/PD-1 pathway is an attractive strategy for cancer treatment; however, the therapeutic effectiveness of PD-L1/PD-1 remains poor (28, 29). Despite the lack of statistical significance, our data suggest a potential trend of elevated PD-L1 expression in the TBA (+) group compared with TBA (−) patients. Further study of the mechanism of the influence of neuronal antibodies on PD-1 will help improve the therapeutic effect of targeted PD-L1/PD-1 immunotherapy.

Recent studies have identified tumor-associated neurogenesis as a potential hallmark of cancer. Similar to how tumors induce angiogenesis to sustain growth, they may also promote the formation of new nerve fibers within the TME. This process involves complex signaling between tumor cells and the nervous system, supporting cancer cell survival and proliferation (30). Nervous system regulation of the gastrointestinal system becomes particularly important in gastrointestinal cancers, in which local nerves affect gastrointestinal tumorigenesis and growth (10). Serum from anti-NMDAR IgA-positive patients, but not from control individuals, has been found to have significant changes in NMDAR-mediated currents (31). Therefore, we speculate that neuronal antibodies affect cancer progression and metastasis by affecting nervous system activity, which warrants further in-depth study.

The insights gained from studying neuronal antibodies and their interactions with cancer have potential therapeutic implications (32, 33). Modulating the nervous system’s influence on tumors may present novel strategies for cancer treatment. Neuromodulation, using genetic and pharmacologic approaches, has shown promise in altering tumor growth and enhancing the antitumor immune response. Additionally, repurposing drugs used for neurologic disorders to target tumor-associated nerves could provide potential effective therapeutic options (34, 35).

### Limitations

This study has several limitations. First, the heterogeneity of neuronal autoantibody responses among patients with gastric cancer poses a challenge to generalizing the results, and the low positive rate of known antibodies in the CBA-tested subset may generate some bias to the result. Second, the single-center design may also introduce selection bias, limiting the generalizability of the results to other populations or settings. Meanwhile, the single-cell data only contain four patients, these findings should be considered hypothesis-generating. Finally, whereas the study identified an association between neuronal autoantibodies and clinical outcomes, the underlying mechanisms through which these antibodies influence tumor progression and immune response remain unclear. Further studies, with larger sample sizes and standardized methodologies, are needed to validate these findings and explore the potential therapeutic implications of neuronal antibodies in gastric cancer.

### Conclusion

In this cohort study, a high prevalence of neuronal autoantibodies was detected among patients with gastric cancer without diagnosed PNS, and neuronal autoantibodies were associated with shortened OS as well as the decreased CD8^+^ T cells in the TME. These autoantibodies might represent a cross-talk between cancer and the neuroimmune system, potentially leading to resistance to immunotherapy.

## Supplementary Material

Supplementary Methods Supplementary Methods

## Data Availability

The single-cell RNA sequencing data generated in this study have been uploaded to Zenodo with designated DOI 10.5281/zenodo.17109917. Other data that support the findings of this study are available from the corresponding author upon reasonable request.
