# Supplementary material for: Association of Neuronal Autoantibodies with Overall Survival in Patients with Gastric Cancer
Source: Cancer Res Commun. 2025 Dec 3;5(12):2102–11. doi: 10.1158/2767-9764.CRC-25-0495 (PMC12673196; doi:10.1158/2767-9764.CRC-25-0495)
Supplement: Supplementary Methods [file crc-25-0495_supplementary_methods_suppsm.docx]

**1. Single-cell methods in this research**

**Sample Preparation**

Fresh tissue specimens were immediately processed to obtain single-cell suspensions. Briefly, tissues were rinsed with DPBS (PBS without calcium and magnesium) and finely minced into small fragments. Enzymatic digestion was performed at 37 °C for 15–25 min using collagenase II (2 mg/mL), collagenase IV (2 mg/mL), and dispase (0.2 mg/mL). The digested material was filtered through a 40 µm nylon mesh (Greiner Bio-One GmbH, Germany) and transferred into centrifuge tubes. Filtrates were centrifuged at 500 g for 5 min, and the supernatant was carefully discarded. Cell pellets were resuspended and treated with RBC lysis buffer on ice for 5 min to remove erythrocytes, followed by two washes with DPBS (500 g, 5 min each). Cell concentration and viability were assessed using a Cellometer Auto 2000 (Nexcelom, USA) after AO/PI staining.

**Library Construction and Sequencing**

Single-cell libraries were prepared following the manufacturer’s protocol for the Chromium Next GEM Single Cell 3ʹ Reagent Kits v3.1 (10x Genomics). Briefly, cell suspensions were adjusted to a concentration of 1,000 cells/μL and loaded onto the Chromium Controller to generate Gel Beads-in-Emulsions (GEMs). Within each GEM, individual cells were encapsulated together with gel beads coated with primers containing a 10x cell barcode, a unique molecular identifier (UMI), and a poly(dT) sequence. Reverse transcription was carried out in GEMs on a Veriti 96-well thermal cycler (Thermo Fisher Scientific, Waltham, MA, USA), thereby incorporating cell-specific barcodes into the synthesized cDNA. Following reverse transcription, cDNA libraries were amplified, fragmented, end-repaired, and A-tailed. Adapters were ligated after size selection, and sample index PCR was performed. Final purification was carried out using SPRIselect beads. Sequencing was performed on an Illumina NovaSeq 6000 platform with paired-end 150 bp reads, generating approximately 500 million reads per library.

**Data Pre-processing and Quality Control**

Raw FASTQ files were processed with Cell Ranger (v7.1.0) against the GRCh38-3.0.0 reference for read alignment, barcode assignment, and UMI counting. Gene expression matrices were imported into Seurat using the Read10X() function. Cells were retained if they met all of the following criteria: 100 < nFeature_RNA < 2,000, nCount_RNA < 10,000, and percent.mt < 20%. These thresholds removed low-quality cells with poor transcript complexity, potential doublets/multiplets with inflated counts, and cells with high mitochondrial content indicative of stress or apoptosis.

**Dimensionality Reduction and Clustering**

Single-cell transcriptomes from different samples were integrated using canonical correlation analysis (CCA). Dimensionality reduction was performed with principal component analysis (PCA) on the top variable genes, and the first 30 PCs were used for Uniform Manifold Approximation and Projection (UMAP). A shared nearest neighbor (SNN) graph was constructed with FindNeighbors (20 PCs), and clusters were identified with FindClusters at a resolution of 0.5. Marker genes were identified using FindAllMarkers (RNA assay), selecting genes with logFC ≥ 0.25 and expressed in ≥25% of cells; the top 10 markers per cluster were visualized to guide cell type annotation. Major cell types were subset, re-clustered at a resolution of 1, and their subcluster-specific markers assessed with FindAllMarkers to determine whether further subdivision or merging was warranted.

**Identification of Differentially Expressed Genes and Pathway Enrichments**

Differentially expressed genes (DEGs) were identified using Seurat’s FindAllMarkers (Wilcoxon rank-sum test, min.pct = 0.25, logfc.threshold = 0.25). Genes with adjusted p < 0.05 were considered significant. Functional enrichment of DEGs was performed with clusterProfiler (v4.0.5). Gene Ontology (GO) enrichment applied Benjamini–Hochberg correction (p < 0.01, q < 0.05), and the top three enriched pathways per cluster were visualized to highlight key biological processes.

**Data Availability Statement**

The single cell RNA-seq data generated in this study has been uploaded to zenodo with designated doi 10.5281/zenodo.17109917.

<https://zenodo.org/records/17109917>

**2. Immunohistochemical (IHC) Staining and Evaluation**

PD-L1 expression was detected via immunohistochemistry (IHC), IHC was performed on 4-mm-thick tissue sections using an automated IHC stainer (Ventana, Tucson, AZ, USA). The assessment of PD-L1 protein expression in GC is a qualitative immunohistochemical assay that uses anti-PD-L1 antibodies (Dako, 22C3) to detect PD-L1 protein in formalin-fixed, paraffin-embedded (FFPE) tissues from gastric adenocarcinomas. A minimum of 100 tumor cells must be present in the PD-L1-stained slide for the specimen to be considered adequate for PD-L1 evaluation. A specimen is considered to have PD-L1 expression if CPS≥1. CPS is the total number of positively stained PD-L1 cells (i.e., tumor cells, lymphocytes, and macrophages) divided by the total number of viable tumor cells, multiplied by 100%. For the patients with CPS in both biopsy and postoperative samples, the final CPS was decided by the higher scores. And the CPS categories in this study were classified as CPS≥5 and CPS<5.

HER2 expression was assessed by IHC and fluorescence in situ hybridization (FISH). IHC was performed using a rabbit monoclonal antibody (clone 4B5; Ventana, Tucson, AZ, USA) on a BenchMark XT automated staining system (Ventana, Tucson, AZ, USA) and scored on a 0 to 3+ scale. FISH was performed using the HER2 DNA Probe Kit (Wuhan HealthCare Biotechnology Co., Ltd., Wuhan, China) and categorized as either positive or negative. The interpretation of IHC and FISH results was based on the CAP/ASCP/ASCO guideline.
